# Disarming the guarded prognosis: predicting survival in newly referred patients with incurable cancer

**DOI:** 10.1038/sj.bjc.6602908

**Published:** 2005-12-13

**Authors:** M R Stockler, M H N Tattersall, M J Boyer, S J Clarke, P J Beale, R J Simes

**Affiliations:** 1 Department of Medical Oncology, Sydney Cancer Centre, Royal Prince Alfred Hospital, Camperdown, NSW 2050 Australia; 2 Department of Medicine, University of Sydney, NSW 2006, Australia; 3 NHMRC Clinical Trials Centre, School of Public Health, University of Sydney, NSW 2006, Australia

**Keywords:** prognosis, communication, truth disclosure, physician-patient relations, survival analysis

## Abstract

People affected by cancer want information about their prognosis but clinicians have trouble estimating and talking about it. We sought to determine the nature and accuracy of medical oncologists' estimates of life expectancy in newly referred patients with incurable cancer. With reference to each patient, medical oncologists estimated how long they thought 90, 50, and 10% of similar patients would live. These proportions were chosen to reflect worst case, predicted, and best case scenarios suitable for discussions. After a median follow-up of 35 months, 86 of the 102 patients had died with an observed median survival of 12 months. Oncologists' estimates of each patient's worst case, predicted and best case scenarios were well-calibrated: 10% of patients lived for fewer months than estimated for the worst 10% of similar patients; 50% lived for at least as long as estimated for 50% of similar patients (predicted survival), and 17% lived for more months than estimated for the best 10% of similar patients. Oncologists' estimates of each patient's predicted survival were imprecise: 29% were within 0.67–1.33 times the patient's actual survival, 35% were too optimistic (>1.33 times the actual survival), and 39% were too pessimistic (<0.67 times the actual survival). The proportions of patients with actual survival times bounded by simple multiples of their predicted survival were as follows: 61% between half to double their predicted, 6% at least three to four times their predicted, and 4% no more than 1/6 of their predicted; similar to the proportions in an exponential distribution (about 50%, 10% and 10% respectively). Ranges based on simple multiples of the predicted survival time appropriately convey prognosis and its uncertainty in newly referred people with incurable cancer.

Most doctors in Western countries now tell patients their diagnosis of cancer, but information about prognosis is less commonly presented. We surveyed 187 people with breast cancer or melanoma and found that only 27% reported a discussion of prognosis around the time of their initial diagnosis (an average of 4 years earlier) (Schofield *et al*, 2001). In a subsequent audio-tape audit of initial consultations with an oncologist in 118 patients with incurable cancer, we found that about half were given some information about life expectancy but only one-third were given a quantified estimate (Gattellari *et al*, 2002). We developed and tested a question prompt list designed to improve communication when cancer patients see a medical or radiation oncologist for the first time. In three separate randomised trials of this intervention, prognosis was the only topic about which patients who received the question prompt list asked more questions, even though only two of the 17 suggested questions were about prognosis (Butow *et al*, 1994; Brown *et al*, 1999, 2001). This failure to discuss prognosis is probably caused as much by doctors' uncertainty about how to think and talk about prognosis as it is by patients' reluctance to ask about it (Brown *et al*, 1999, 2001). Christakis and Lamont (2000) reported that doctors were inaccurate in their estimates of prognosis for terminally ill patients, and that their errors were systematically optimistic. In their cohort of 468 patients whose median survival was 24 days, only 20% of survival predictions met their criterion of accuracy (predicted survival within ±33% of actual survival); 63% were too optimistic and 17% were too pessimistic. Lamont and Christakis (2003) have also suggested that physicians may have difficulty finding the research data they need to better estimate the survival of their patients with advanced cancer.

The aim of this study was to determine the nature and accuracy of medical oncologists' predictions of survival in newly referred people with incurable cancer.

## PARTICIPANTS AND METHODS

Baseline characteristics (age, primary site, histology, date of diagnosis, stage at diagnosis, current extent), prior treatment, and planned treatment were recorded prospectively for all outpatients referred to any one of the 11 medical oncologists at the Royal Prince Alfred Hospital campus of the Sydney Cancer Centre, NSW, Australia. Oncologists also recorded whether the intent of future treatment was curative or noncurative. For patients in whom the intent of treatment was noncurative, oncologists were asked to complete three additional questions headed ‘Estimation of prognosis’ as follows:

‘If a group of patients had a similar stage of cancer and prognostic factors…
What is the length of time (months) 90% would survive?—;
What is the length of time (months) 50% would survive (i.e. median survival)? —;
What is the length of time (months) the best 10% would survive?—.

There was no further specification of what ‘similar stage of cancer and prognostic factors’ meant. Participating oncologists understood that the ‘length of time 50% would survive (i.e. median survival)’ was asking for the oncologist's best estimate of how long that individual patient would live. This estimate of life expectancy is referred to as the ‘predicted survival’ in this paper. These questions were designed to elicit predictions that might be used in discussions with patients to reflect worst case, typical, and best-case scenarios.

All data were recorded on a single page form that is completed as part of routine clinical practice for all patients referred to our unit, generally within a week or two of their first consultation, after any additional tests had been completed. The study was considered an audit of standard practice. It involved no additional questions, tests, or interventions for patients, had no effect on their medical care, and did not involve any external research personnel. Ethics clearance and patient consent are not required for such audits in Australia.

The sampling frame for this study consisted of 500 consecutive newly referred outpatients seen over a 5-month period. Estimates of prognosis were available for 102 of the 205 patients in whom the intent of treatment was considered noncurative (response-rate of 50%). We did not try to determine why estimates of prognosis were not recorded for the other 50% of patients with incurable cancer. Just over half were referred by surgeons, approximately 15% by local doctors, 11% by physicians, and the remainder by gynaecologists or radiation oncologists.

The objectives of the analysis were descriptive and exploratory. Durations of survival and follow-up are described with the Kaplan–Meier product-limit method and are based on all 102 patients. Comparisons of predicted survival with observed survival include all 102 patients. The findings and conclusions were unaffected by excluding patients who were alive at the last follow-up.

## RESULTS

The patients' baseline characteristics are summarized in Table 1 and are typical of people with advanced cancer newly referred to our centre. Most were symptomatic, older than 50 years, and had some form of anticancer treatment recommended.

**Figure 1:**
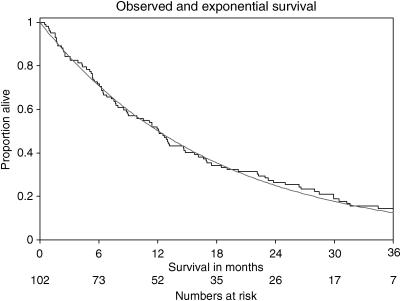
Observed survival distribution (step function) and an exponential distribution based on a median survival of 12 months (smooth curve).

After a median follow-up of 35 months, 86 of the 102 died. Their survival distribution is shown in Figure 1. The median survival of the cohort was 12 months (range 2 weeks–38 months). The figure illustrates that the observed survival distribution was closely approximated by an exponential distribution with a median survival of 12 months.

Oncologists' predictions were well-calibrated. Half the patients (50%) lived at least as long as their oncologist's ‘predicted survival’ (the number of months 50% of similar patients would survive); 10% lived fewer months than their oncologist predicted for the worst 10% of similar patients; 17% lived more months than their oncologist predicted for the best 10% of similar patients. There was a strong correlation between the oncologists' predicted survival and their patients' actual survival (Spearman's rank correlation *r*=0.60, *P*<10^−6^).

**Figure 2:**
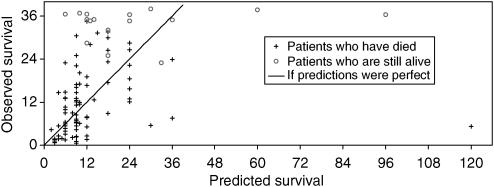
Observed versus predicted survival for each individual. Points on the 45 degree line signify people who lived exactly as long as predicted, points above the line signify people who lived longer than predicted, points below the line signify people who lived shorter than predicted. There is a strong association between predicted and observed survival (Spearman's rank correlation of 0.60, *P*<10^−6^).

**Figure 3:**
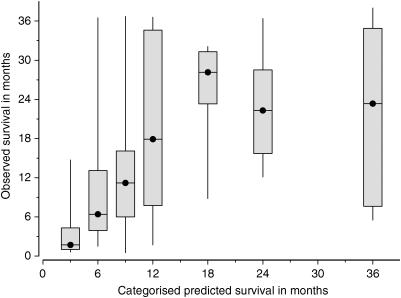
Observed survival for subgroups with similar predicted survivals. The categories (range of included predictions, and number of patients) are: 3 months (2–4, *n*=10); 6 months (5–7, *n*=17); 9 months (8–10, *n*=31); 12 months (11–14, *n*=16); 18 months (15–20, *n*=10); 24 months (21–29, *n*=9); 36 months (>29, *n*=9). The boxes extend from the upper quartile (75th percentile) to the lower quartile (25th percentile) and include the middle 50% of patients. The solid circle represents the median (50th percentile). The whiskers extend to the maximum and minimum value.

Oncologists' predictions were imprecise. Figure 2 illustrates the relationship between the oncologists' predicted survival for each patient and their actual survival. Most predicted survivals were simple multiples of 3 or 4 months (e.g. 3, 4, 6, 8, 9, 12, 18 and 24 months). Figure 3 shows the actual survivals for subgroups of patients with similar predicted survivals. The range of actual survivals within each subgroup is wide and skewed towards longer times. The actual median survival for each subgroup is close to that predicted.

Few patients had actual survival times close to their oncologist's predicted survival. In all, one-twentieth (5%) lived within a month of their oncologist's predicted survival, one-sixth (18%) lived within 2 months of it, and one-third (32%) lived within 3 months of it. Only 29% of oncologists' predicted survivals met Christakis' criterion of accuracy by falling within 33% of the patient's actual survival (i.e. predicted survival between 0.67 and 1.33 times the actual survival). Similar proportions of the predictions were either too optimistic (35% of predictions more than 1.33 times the actual survival) or too pessimistic (39% of predictions <0.67 times the actual survival).

The proportions of patients with actual survival times bounded by simple multiples of their predicted survival time were similar to those expected in an exponential distribution. About one-third of patients (35%) lived between 0.67 and 1.5 times their predicted survival time, and about half (61%) lived between half to double their predicted survival time, 6% lived for at least three to four times their predicted survival time, and 4% lived for no more than 1/6 of their predicted survival time. These proportions are similar to those bounded by the multiples of the median survival in an exponential distribution: 28% of values between 0.67 to 1.5 times the median, and 46% between half to double the median, 10% more than three to four times the median, and 10% <1/6 of the median.

## DISCUSSION

Oncologists' predictions were well-calibrated but imprecise. Few patients had actual survival times close to their oncologist's prediction, but there was no systematic tendency for oncologists to either overestimate or underestimate, and substantial proportions of patients lived within simple multiples of their oncologist's predictions. The strengths, limitations and implications of these observations are discussed below.

The strength of this study is its prospective design and follow-up. Survival predictions were made at or near the initial consultation. Only 16 people were still alive at the time of our analysis. This biases our estimates of actual survival downwards, but has little effect on estimates of median survival (either for all patients, or for subgroups). Including or excluding these people from the analyses had little effect on the findings. However, an analysis performed after these 16 people die would be likely to show that oncologists underestimated the survival times of those who lived longest.

The main limitations of this study are its size and response rate. There are too few patients and oncologists to draw conclusions about subgroups. The accuracy of our predictions is probably overestimated because the prognosis in patients for whom predictions were recorded was probably more straightforward than that in patients for whom predictions were not recorded. However, the group's median survival of 12 months is almost identical to that of complete cohorts of our patients with incurable cancer from 1977 to 1993 (Milsted *et al*, 1980; Chye *et al*, 1984). Our results are probably best considered to reflect a group of newly referred patients with incurable cancer for whom oncologists were willing to record estimates of prognosis.

Most previous studies of prognostication in incurable cancer have been in people with far-advanced disease being referred for end-of-life care, not in people recently diagnosed and being referred to medical oncologists for consideration of anticancer treatment. These studies have shown that doctors' predictions were inaccurate, with a tendency to overestimate life expectancy (Vigano *et al*, 1999, 2000a, 2000b; Christakis and Lamont, 2000). People in these previous studies were being admitted to hospices or hospice programmes and most died within a few weeks or months. Christakis and Lamont (2000) defined predictions that were within ±33% of the observed survival as accurate, predictions <0.67 times the observed survival as too pessimistic, and predictions >1.33 times the observed survival as too optimistic. In their study of 468 terminally ill people referred to an outpatient hospice programme with a median survival of 24 days, 20% of predictions made by referring clinicians' were accurate, 63% were too optimistic, and 17% were too pessimistic. In our study, a higher proportion of predictions were accurate (29%), while similar proportions were either too optimistic (35%) or too pessimistic (39%). MacKillop and Quirt's (1997) findings in their study of 39 patients with advanced cancer and a median survival of 14 months are similar to ours: 31% of oncologists' predictions were accurate, 38% were too optimistic and 31% were too pessimistic. The lower accuracy and tendency to overestimate life expectancy in Christakis and Lamont's study probably reflects their population's shorter survival, and the large number of generalist physicians less familiar with advanced cancer.

The distribution of our group's actual survival times was skewed to the right (towards longer times), as are most survival distributions. This is because the minimum survival time can be no shorter than 0, whereas the maximum survival time can be many years. The same constraints should apply to estimates for an individual. Someone with a predicted survival of 6 months can die no sooner than immediately, but may live for several years. This suggests that if ranges are to be estimated around a predicted survival, then they should also be asymmetrical – the interval above the predicted survival should be larger than the interval below it.

The good fit of an exponential model was fortuitous (the first and only fit we tried) and surprising because our population included a mixture of types and extents of advanced cancer with different expected survival durations. We are not suggesting that the survival distributions of all groups of cancer patients are exactly exponential. More homogeneous groups should have survival curves that are sigmoidal (steeper in the middle, flatter at the beginning and end); more heterogeneous groups should have survival curves better approximated by a declining exponential (steeper at the beginning and flatter at the end). However, keeping the exponential shape in mind is helpful in thinking and talking about predictions of life expectancy, even if it does not provide an exact fit.

The median survival is the time taken for a group to be halved (half still alive, half already dead), and in an exponential distribution, this time is constant along the whole curve and analogous to the half-life of radioactive decay. So, in an exponential distribution the proportion remaining after two half-lives is 25%, after three half-lives is 12.5%, and after four half-lives is 6.25%.

These observations have important implications for how we might think and talk about predicted life expectancy. Firstly, the predictions were well-calibrated, so predicting the median survival of a group of similar patients seems a reasonable starting point. Secondly, predictions were imprecise and probabilistic, so it is probably better to think and talk about ranges (e.g. 6–18 months) than about single point estimates (e.g. 9 months). Thirdly, survival times are skewed to the right (towards longer times), so ranges around any point estimate (for example the predicted median survival) should be asymmetrical with wider intervals above than below. Fourthly, it is helpful to think of median survivals as half-lives and to use simple multiples of the predicted median survival (e.g. half to double) to construct ranges. This corresponds with Lamont and Christakis' recommendation to discuss the interquartile range – half the median estimates the upper quartile (75% live at least this long), and double the median estimates the lower quartile (25% live at least this long) (Lamont and Christakis, 2003). Lastly, rough predictions of the best and worst case scenarios might be estimated as about three to four times, and 1/6 of the predicted median, respectively.

The stereotypical question about life expectancy – ‘How long have I got?’ – begs a simple, single-number answer. Unfortunately, the stereotypical, simple, single-number answer – ‘6 months’ for example – is as misleading as it is unsympathetic. A single number suggests greater precision than is warranted, and is often interpreted as a limit – ‘the last doctor said I had only 6 months, but that was a year ago and I'm still going strong’. We suggest it may be better to think and talk about ranges based on an exponential model. Box 1 outlines the suggested steps for predicting life expectancy in people with advanced cancer using this approach. We recommend deliberately leaving estimates rough to accurately convey their inherent imprecision.

Before discussing estimates of life expectancy with an individual, it is important to determine what kind of information they want. Do they want any information at all, and if so, would they prefer orders of magnitude (e.g. days to weeks, weeks to months, months to years); lengths of time (e.g. numbers of days, weeks, months or years); or probabilities (e.g. the chance of living a given length of time)? Box 2 gives examples of how estimates of life expectancy might be discussed and explained, depending on the patient's information preferences.

Our data do not indicate how to improve the accuracy of individual predictions. We asked oncologists to estimate ‘how many months 50% of similar patients would survive’; asking them ‘how many months do you think this patient will survive’ might have resulted in different answers. There was a strong correlation between oncologists' predictions and their patients' actual survival times. However, over half the variation in patients' survival times remained unexplained. The prognostic importance of performance status and quality of life are well documented in advanced cancer (Stockler *et al*, 1999; Chow *et al*, 2002). Symptoms and signs of advanced cancer, nutritional status, and laboratory tests have also been identified as important (Maltoni and Amadori, 2002). At the time of initial referral to a medical oncologist, other factors may also be important, such as disease tempo, response to previous treatments, co-morbidities, and planned future treatments. Better understanding of these factors and their significance should help doctors refine and improve the accuracy of their predictions. However, it may be that life expectancy, like many other complex phenomena, is inherently unpredictable and the best we can do is improve our appreciation and communication of this uncertainty.

Estimates of life expectancy are essential for rational decision-making, planning and management in people with advanced cancer, many of whom want more information about their prognosis. Describing life expectancy with approximate ranges based on simple multiples of the predicted median survival of a group of similar patients appropriately conveyed prognosis and its uncertainty in newly referred patients with advanced cancer. An appreciation of life's inherent unpredictability and how to describe it should help clinicians better meet the needs of those affected by advanced cancer.

## Figures and Tables

**Table 1:** Baseline characteristics (*n*=102)

*Age in years*	
Median	64
Interquartile range	55–73
Range	16–96
	
Female (%)	41
	
*Tumour type* (%)	
Lung	18
Colorectal	11
Breast	10
Carcinoma of unkown primary site	9
Melanoma	8
Prostate	7
Liver	5
Kidney	4
Ovary	4
Bladder	3
Stomach	3
Other	26
	
*Previous anticancer treatment* (%)	
Surgery	59
Hormone therapy	13
Radiation therapy	13
Chemotherapy	7
None	34
	
Symptomatic (%)	73
	
*Type of treatment planned* (%)	
Chemo	57
Radiation	22
Hormonal	45
Observation	10
Surgery	1
Other	4
	
*Nature of treatment planned* (%)	
Standard	62
Clinical Trial	15
Other	14
None	9

**Box 1:** 

Steps for predicting life expectancy
1. Estimate the median survival of a group with similar characteristics
2. Adjust the median survival from the group to account for differences with the individual
3. Estimate the range for the middle half of patients by taking half to double the predicted median
4. Estimate the best and worst case scenarios as ∼1/6 of, and three to four times, the predicted median
5. Adjust the best and worst case scenario estimates to account for any outstanding differences or biases

**Box 2:** 

*Examples of phrases for talking about life expectancy*
1. The typical person with your type and stage of cancer lives *X* months. This means that half the people live more than *X* months and half the people live less than *X* months
2. About half the people with your type and stage of cancer live between *X*/2 and 2*X* months
3. If we had 100 people exactly like you, then we did expect that the 10 who did best to still be around in 3–4*X* months, whereas the 10 who did worst would be in trouble within *X*/6 months
4. It might be as short as a few months or as long as a few years
